# Ureteric Injury During Gynaecological Surgery – Lessons from 20 Cases in Canada

**Published:** 2020-05-07

**Authors:** GP Jacob, GA Vilos, F Al Turki, G Bhangav, B Abu-Rafea, AG Vilos, A Ternamian

**Affiliations:** Department of Obstetrics and Gynecology, Chatham-Kent Health Alliance, Chatham, Ontario, Canada;; Department of Obstetrics and Gynecology, Western University, London, Ontario, Canada;; Department of Obstetrics and Gynecology, King Fahad Medical City, Riyadh, Saudi Arabia;; Department of Obstetrics and Gynecology, University of Toronto, Toronto, Ontario, Canada.

**Keywords:** Ureteral injury, gynaecological surgery, litigated ureteral injury, urinary tract injury

## Abstract

**Background:**

Ureteric injury is a complication of gynaecological surgery that can cause significant morbidity for the patient and is a leading cause of litigation in many countries.

**Objectives:**

To determine patient characteristics, peri-operative circumstances and clinical and legal outcomes of ureteral injuries associated with gynaecological surgery.

**Patients and methods:**

This is a retrospective review of 20 cases of ureteric injury during benign gynaecological surgery.

**Main outcome measures:**

All cases were assessed for the following variables–patient characteristics, indications for surgery, injury, postoperative symptoms and presentation, and clinical and legal outcomes.

**Results:**

Risk factors associated with ureteric injury included obesity, previous laparotomic pelvic surgery, pelvic adhesions, large pelvic masses and intra-operative bleeding. 70% (14/20) of ureteral injuries were diagnosed after discharge. 50% (10/20) of patients had a complicated post-operative course and 45% (9/20) of cases resulted in unfavourable legal outcomes (settlement or lost at trial) for the surgeon. The conduct of surgery and the failure to act in a timely fashion postoperatively were the most frequent reasons for adverse clinical and unfavourable litigation outcomes for the surgeon.

**Conclusions:**

Intra-operative surgical consultation and ureteral identification should be considered if there is concern for ureteral involvement in the surgical field. Ureteric injury may not constitute negligence if it is demonstrated that the surgeon provided reasonable care that would be expected during the peri-operative phases.

**What is new:**

This review identifies patient characteristics and peri-operative variables that correlate with poor clinical and legal outcomes after ureteric injury.

## Introduction

The ureter is at risk of injury during any gynaecological surgery not only because of its close proximity to pelvic organs such as the rectosigmoid and the utero-cervical junction, but also due to the biological variability of its location, as it travels from the kidney under the ovarian vessels, crosses over the external iliac arteries and the pelvic brim to travel loosely attached to the medial leaf of the pelvic side- wall peritoneum, travels under the uterine arteries and tunnel through the cardinal ligament and upper anterior vagina to enter the bladder. The ureter is most vulnerable to injury during several steps of pelvic surgery including the level of the pelvic brim when the ovarian vessels are secured and transected, during the securing and transection of the uterine arteries, near the ureterovesical junction during the dissection of the bladder from the cervix and upper vagina, or during the closure of the vaginal cuff following hysterectomy.

Mechanisms of ureteral injuries include contusion, devascularisation, kinking, laceration, application of clips, suture-ligation, and transection. Following the introduction of laparoscopic pelvic surgery including hysterectomy, thermal injuries by inadvertent direct application or by thermal spread of heat to the ureter from the various sources of energy used (radiofrequency, ultrasonic, etc.) have emerged as another significant mechanism of injury.

Ureteric injury is a serious complication of pelvic surgery with a reported rate for hysterectomies that varies from 0.02% to 0.78% (Gilmour et al., 2006). Ureteral injuries are associated with all methods of hysterectomy, irrespective of the method (abdominal, vaginal, or laparoscopic). The rate of ureteral injury in a large prospective Finnish study of 5279 hysterectomies was 0.3%, 0.04%, and 0.3% for the abdominal, vaginal, and laparoscopic approaches, respectively (Brummer et al., 2011).

A 2018 systematic review including 433 studies representing 140,444 gynaecologic laparoscopic surgeries for benign indications, reported 458 lower urinary tract injuries for an incidence of 0.33%. Bladder injury (0.24%) was overall three times more frequent than ureteral injury (0.08%) (Wong et al., 2018). In a retrospective review involving 3114 hysterectomies, the rate of ureteral injury of robotic hysterectomy (7/1088, 0.64%) was similar to laparoscopic (4/782, 0.51%), vaginal (1/304, 0.33%) and abdominal hysterectomy (5/940, 0.53%) (Petersen et al, 2018).

Predisposing factors such as abnormal pelvic anatomy, endometriosis, pelvic adhesions, large adnexal masses, and unexpected intraoperative bleeding, have been associated with an increased likelihood of ureteral injury (CMPA, 2015). In the 2018 systematic review of urinary tract injury in gynaecological laparoscopy for benign indications, the highest incidence of ureteral injury was found in endometriosis resection (0.4%, 95% CI 0.3– 0.6) followed by laparoscopically assisted vaginal hysterectomy (0.2%, 95% CI 0.2–0.3) and laparoscopic hysterectomy not otherwise specified (0.2%, 95% CI 0.1–0.6) (Brummer et al, 2011). However, in over 40% of all ureteric injuries, there are no predisposing factors that can be identified, and the surgery performed is described as routine (Härkki-Sirén et al., 1998).

Ureteric injury can lead to significant patient morbidity including an irreversible loss of renal function leading to chronic renal failure and/or the loss of a kidney. This is particularly true when the injury is not recognized and acted upon in a timely fashion. Consequently, ureteral injury is invariably perceived by patients and their families as substandard surgical care which provokes potential or actual ligation. In fact, a review of urinary tract injuries in Canada reported that the risk of litigation after a ureteric injury was quite high (91% relative risk) even though it was a rare complication (< 1%) (Gilmour & Baskett, 2005). Urinary tract injury has also been reported to be the most common cause of litigation after gynaecological surgery in other countries including Denmark, Saudi Arabia and Holland (Hove et al., 2010; AlDakhil, 2016; Sandberg et al., 2017).

Given the significant clinical and legal implications of inadvertent ureteric injury, we reviewed and summarized 20 cases of ureteral injuries to highlight several issues including patient characteristics, pre-operative care, predisposing factors to ureteral injury, intra-operative management, postoperative care, and clinical and legal outcomes.

## Materials and methods

From 1988 through to 2015, we reviewed 20 cases of ureteric injury associated with routine gynaecological surgery in Canada. Some of these cases (six) were litigated and concluded by the courts and are in the public domain. The majority of the cases (fourteen) were referred to the authors for expert review as part of potential or ongoing litigation. Appropriate cases were reviewed and reconstructed by altering recognizable patient and surgeon indicators to highlight the patient characteristics, predisposing factors, peri-operative circumstances and clinical and legal outcomes of ureteral injuries. Consequently, we feel that no institutional review and ethics approval was required.

The variables examined included indications for any type of surgery, intra-operative care, post- operative presentation and intervention and clinical and legal outcomes. Cases were selected if there was sufficient clinical and/or legal information to complete the assessment. Complicated clinical outcome was defined as prolonged care in the intensive care unit, physical impairment or death.

## Results

### Ureteral injury associated with vaginal hysterectomy

Table I summarizes 2 cases of ureteral injuries associated with vaginal hysterectomy and anterior and posterior repair.

**Table I:** Patient characteristics and ureteral injuries associated with Vaginal Hysterectomy (VH-vaginal hysterectomy, A&P-anterior and posterior AUB-Abnormal uterine bleeding, POD- Post operative day, IVP- Intravenous pyelogram, US- Ultrasound, WBC-white blood cell.

Case/Year	Age/parity	BMI	Comorbidities	Indications for surgery	Surgery/Hemostatic method used	Presentation	Investigation/Intervention	Legal outcome
1/1993	63 G2P2	NA	none	Urinary stress incontinence, uterine prolapse and first degree urethro-cystocele.	VH with A & P repair. Clamp, cut and suture ligate	POD #3-5: Back pain and left abdominal and flank pain	POD #7: Severe left flank pain. US: Left hydronephrosis, ureteral obstruction. Percutaneous nephrostomy and double J-stent. Uneventful resolution	Abandoned
2/2001	35 G2P2	NA	None	AUB, dysmenorrhea, dyspareunia and pelvic organ prolapse with moderate cystocele and rectocele	VH with A & P repair. Clamp, cut and suture ligate	POD #1-2: Back and right flank pain, nausea, Temperature 38.6° C x 2 WBC was 12 X 10 9 /L	POD #2: IVP: Right ureteral obstruction at vaginal cuff. Laparotomy: Reimplantation, uneventful resolution	Abandoned

### Ureteral injury associated with laparoscopic hysterectomy

Table II summarizes 9 cases of ureteral injuries associated with laparoscopic assisted vaginal hysterectomy (LAVH) and one case with laparoscopic supra-cervical hysterectomy (LSH).

**Table II:** Patient characteristics and ureteral injuries associated with laparoscopic hysterectomy. (BMI- Body mass index, NA-Not available, AUB-Abnormal uterine bleeding, LAVH-Laparoscopic assisted vaginal hysterectomy, LSH- Laparoscopic subtotal hysterectomy, RSO-Right salpingo-oophorectomy, LSO- Left salpingo-oophorectomy, POD- Post operative day, IVP- Intravenous pyelogram, US- Ultrasound, CPP- Chronic pelvic pain, LT-left).

Case/Year	Age/parity	BMI	Comorbidity	Indications for surgery	Surgery/Energy source used	Presentation	Investigation/Intervention	Legal outcome
3/1995	26 G1P1	NA	Anemia	AUB, Fibroids	LAVH/Stapling device POD #2: Discharged	6-8 weeks: Low back pain 9 weeks: Lt hydrosalpinx; 3-months: Laparoscopy RSO	4-months post LAVH: IVP-Lt ureteral obstruction: percutaneous nephrostomy. Reimplantation, resolution.	Dismissed
4/1996	40 G3P3	39	Anemia, Hypertension, Asthma	AUB	LAVH/Stapling device. Bleeding right uterine vessel sutured vaginally. Estimated blood loss 900mL.	POD #6-9: Worsening abdominal pain, fever, urine per vagina. POD #6: US-Ileus	POD #9: IVP-Rt distal ureteral transection, extravasation of urine-Nephrostomy. POD #17: Laparotomy, Rt ureter stapled/transected. Reimplantation with psoas hitch	Dismissed
5/1997	48 G2P2	NA	None	AUB Dys-menorrhea, chronic endometritis.	LAVH/Stapling device. POD #1: Discharged	POD #8: Back pain.	POD #8: US & IVP-Rt hydronephrosis, Percutaneous nephrostomy. POD #38: Laparotomy, Rt ureter stapled/transected. Reimplantation with psoas hitch	Dismissed
6/1993	53 G3P3	NA	Anemia	AUB, fibroids	LAVH+BSO/Bipolar electro-surgery. POD #3: Discharged	POD #7: Low back pain, nausea, vomiting, dysuria, hematuria	POD #7: US; Lt ureter obstruction. POD #10: Percutaneous nephrostomy. POD #16: Pyelogram; Rt ureterovaginal fistula- Stents inserted. POD #60: Resolution of injury	Settlement
7/1996	47 G3P2	NA	Two previous laparotomies	AUB	LAVH+LSO/Bipolar electro-surgery	POD #2-5: Nausea, ileus POD #6: Discharged POD #14: Urine per vagina. BUN-18.3mmol/L. Serum creatine-469 micromol/L.	POD #14: IVP-Bilateral hydronephrosis, reduced kidney function, pelvic extravasation Rt ureter, vaginal extravasation Lt ureter. Laparotomy: Bilateral reimplantation	Trial-Negligence. Likely thermal injury.
8/2012	49	NA	None	AUB	LAVH/Bipolar electro-surgery	POD #10-16: Abdominal pain, intermittent urinary retention. US: 16cm hematoma.	POD #16: laparoscopy-no hematoma. CT scan: Left ureteral transection. Percutaneous nephrostomy. 4 months: laparotomy-Reimplantation	Trial-Negligence. Over-turned on appeal
9/2000	41 G5P2	24	None	AUB, Fibroid uterus CPP, Adnexal cyst	LSH/Bipolar electro-surgery POD #2: Discharged	POD #3: Abdominal pain and distention. BUN and creatinine-normal	POD #3: CT scan-Right hydronephrosis, intraperitoneal fluid. Cystoscopy/retrograde pyelogram: Extravasation of urine into abdomen. Double J-stent: resolution of injury.	Settlement
10/2012	32	NA	None	AUB	LAVH/Harmonic Scalpel	POD #2: Abdominal pain, rising serum creatinine.	POD #2: Laparotomy; Rt ureteral transection at cardinal ligament; Reimplantation.	Trial-No negligence
11/2014	52 G0P0	58	None	PMB, Endometrial cancer	LAVH/BSO/bipolar electrosurgery; bleeding/Laparotomy	POD #7 Abdominal pain, elevated serum creatinine.	POD #22: CT: free urine in abdomen, Lt ureteral injury, percutaneous nephrostomy. 1 year: Laparotomy- Reimplantation	Settlement

### Ureteral injury associated with total abdominal hysterectomy with and without salpingo- oophorectomy

Table III summarizes 5 cases.

**Table III:** Ureteral injuries associated with total abdominal hysterectomy +/- oophorectomy (BMI- body mass index, NA- Not available, AUB-Abnormal uterine bleeding, POD- Post operative day, TAH- Total abdominal hysterectomy, LSO- Left salpingo- oophorectomy, RSO- Right salpingo-oophorectomy, BSO- Bilateral salpingo-oophorectomy, IVP- Intravenous pyelography, US- Ultrasound, CPP- Chronic pelvic pain, HTN- hypertension.

Case/Year	Age/Parity	BMI	Comorbidity	Indication for surgery	Surgery	Presentation	Investigation/Intervention	Legal outcome
12/2003	43	35	Obesity	AUB, Large fibroid uterus	TAH 3 hours. Significant bleeding sutured. Ureters- good peristalsis	POD #1: Asymptomatic. US to exclude suspected injury-Rt hydrone-phrosis, ureteral obstruction	POD #1: Percutaneous nephrostomy. 3 months: Laparotomy- reimplantation	Trial-won by Defendant
13/1991	53	40	Obesity, Hysteropexy, Appendectomy, LSO	AUB, Cervical dysplasia	TAH+RSO. Difficult adhesiolysis	POD #0: Oliguria, urine per vagina	POD #0: IVP-Cystotomy, left ureteral obstruction. POD #1: Laparotomy: Bladder repair, ureteral reimplantation. POD #2: Pulmonary embolism, death	Trial-won by defendant
14//1995	47/ G2P1	NA	None	AUB	TAH-BSO. Ureters identified at pelvic brim	POD #1-3: Fever -38.5° C x 2,HTN	POD #42: no issues POD #75: US-Right hydronephrosis. Nephrostomy-failed to recover kidney function. Nephrectomy	Dismissed. Renal failure, likely to pre-existing HTN
15/2000	47 G1P1	NA	Caesarian section, HTN, Migraines	AUB Fibroid uterus	TAH-BSO. Ureters identified at pelvic brim. Bleeding sutured left vaginal vault.	POD #38: Left loin pain	POD#45: IVP/CT/cystoscopy/retrograde pyelography-Lt ureteral obstruction, urine extravasation. Percutaneous nephrostomy Laparotomy: Reimplantation	Dismissed
16/2004	36	NA	Endometriosis Fibroid uterus	AUB Uterine fibroids CPP	TAH-BSO Intraoperative: Left ureteral injury repaired by ureteroneo-cystotomy	POD #5: Intermittent back pain. POD #6: discharged	POD #35: IVP: Right hydronephrosis, hydroureter. Left ureter-normal Percutaneous nephrostomy Laparotomy: Reimplantation with Boari flap	Settlement

### Ureteral injuries associated with laparotomic removal of pelvic cysts

Table IV summarizes 4 cases.

**Table IV:** Ureteral injuries associated with adnexectomy (BMI- Body mass index, NA- Not available, POD- Post operative day, LSO- Left salpingo-oophorectomy, RSO- Right salpingo-oophorectomy, IVP- Intravenous pyelography, US- Ultrasound).

Case/Year	Age	BMI	Comorbidity	Indications	Surgery	Presentation	Investigation/Intervention	Legal outcome
17/1997	45	NA	4 laparotomies including TAH and LSO	Pelvic pain, pressure. 13 cm right ovarian cyst	Laparotomy: Difficult RSO, extensive adhesiolysis. Unable to identify ureter	POD #1-6: Intermittent right flank pain, vomiting. leukocytosis POD #7: Pathology reported a segment of ureter	POD #8: IVP-Rt hydronephrosis, obstructed ureter. Percutaneous nephrostomy. Laparotomy: Ureteroneocystotomy	Settlement
18/1998	49	NA	Obesity, 2 laparotomies	Pelvic pain, 13 cm pelvic cyst	Laparotomy: Retroperitoneal pelvic cyst excision, extensive adhesiolysis. Ureters not identified	POD#2: Smell of urine in abdominal drain POD #7: Uretero-rectal fistula	POD #2: Retrograde pyelogram-Left ureteral injury. Laparotomy: ureteral injury repaired POD #7: Colostomy for ureterorectal fistula	Trial: Negligence for ureter but not for bowel
19/2001	42	55	Obesity, Multiple laparotomies including TAH	Pelvic Pain. 12 cm right ovarian mass	Laparotomy: Right oophorectomy. Ureter identified retroperitoneally.	POD #11: Pelvic pain, back pain, urinary retention	POD #11: IVP/US/Cystoscopy- Right ureteral injury. Percutaneous nephrostomy 2 months: Laparotomy- reimplantation	Dismissed
20/2010	41	46	Previous TAH	Pelvic pain. Bilateral complex ovarian cysts	Laparotomy: Bilateral oophorectomy. Adhesiolysis. Ureters identified.	POD #10: Left abdominal pain	POD #10: IVP/US-Left hydronephrosis, metal clip obstructing left ureter. Percutaneous nephrostomy. 2 months: Laparotomy- Reimplantation using Boari flap	Settlement

## Discussion

In this review, ureteral injuries were associated with all methods of hysterectomy, irrespective of the method (vaginal, laparoscopic, or laparotomic). This is consistent with literature findings. According to a 2006 review, corresponding rates of ureteral injuries for vaginal, laparotomic and laparoscopic hysterectomy were 0.2, 1.3 and 7.8 per 1000 procedures, respectively (Gilmour et al, 2006). Older published reports indicate that the overall incidence of ureteric injury associated with laparoscopic surgery was approximately 1% (Saidi et al., 1996; Tamussino et al., 1998; Ostrzenski et al., 2003; Manoucheri et al., 2012). However, more contemporary publications indicate that the incidence of such ureteric injuries during minimally invasive gynaecological surgery is much lower, ranging from 0.02% to 0.4% (Adelman et al., 2014). During hysterectomy, several steps have been proposed to minimize/avoid the risk of ureteral injury. Specifically, the first step is to identify the ureter at the pelvic brim (retro- or trans- peritoneally) and follow it caudally along the lateral pelvic side wall to where it disappears under the uterine arteries though the broad ligament. This step avoids/minimizes the risk of ureteral injury at the pelvic brim during securing and transecting the infundibulopelvic ligament (ovarian vessels) with sutures or an energy source.

The second step is to incise the round ligament and anterior leaf of the broad ligament and peritoneum overlying the bladder and cervix and to reflect it laterally and downwards below the junction of the uterus and internal os of the cervix. This step displaces the ureter downwards and laterally towards the uterine artery. The third step is to skeletonize the uterine arteries which displaces the ureter further downwards and laterally to the uterine arteries prior to securing and transecting the uterine arteries.

Although these steps are taught and may be practiced routinely during laparotomic hysterectomy, skeletonization of the uterine arteries during laparoscopic hysterectomy may not be practiced routinely due to technical challenges. This might explain the higher rates of ureteral injury associated with laparoscopic hysterectomy reported by the earlier publications.

Although the clinical presentation and outcomes of ureteral injury following any method of hysterectomy may be similar, the legal outcomes appear to be different. In both cases of ureteral obstruction associated with vaginal hysterectomy (Case 1 & 2, Table 1), experts opined that the standard of care was met in the pre-, intra-, and post- operative care, and litigation was not initiated in case 1 and abandoned in case 2. The mechanism of ureteral obstruction in Case No. 1 is consistent with significant ureteral kinking since it declared itself early and it resolved uneventfully after percutaneous nephrostomy and double J-stent. In Case No. 2, the ureter was most likely obstructed by suture.

These two cases indicate that ureteral injury during vaginal hysterectomy in itself is not an indication of breach of the standard of care, provided that the indications for surgery were appropriate, appropriate surgical technique was practiced and the injury is recognized and managed appropriately in a timely fashion.

The 9 cases of laparoscopic hysterectomy (LAVH and LSH, Table II) reflect the utilization of various methods and energy sources used to secure vessels and remove the uterus during the last 30 years. In Case No. 3, it is not clear when and how the ureter was obstructed while in Cases No. 4 and 5 the ureter was clearly stapled across and transected. This type of injury was frequent in the 1990s after stapling devices were introduced for LAVH. The mechanism of this injury was likely due to the larger diameter of the stapling devices (10 mm) and their ‘bite’ included the ureter together with the uterine arteries if the ureter was located less than 1 cm laterally to the cervix.

In cases No. 6 through 9 and No. 11, a bipolar coagulating device was used to secure the vessels while a harmonic scalpel was used in Case No. 10. In all these cases, the most likely mechanism of ureteral injury was thought to be thermal burn by inadvertent direct coagulation of the ureter or by lateral spread of heat to the ureter.

Again, the standard of care was met in all cases regarding pre-operative care. In one case (Case No. 8), the issue of informed consent was raised but was defeated when it was considered that additional information would not have changed the patient’s mind to proceed with the required hysterectomy. Of the 6 litigated cases of ureteral injury associated with laparoscopic hysterectomy and the use of an energy source, 3 cases were settled, one was won by the Plaintiff and two by the Defendant at trial (Table II).

Of the 5 ureteral injury cases associated with TAH +/- BSO (Table III), four legal outcomes were favourable to the Defendant. One case (No.16) involving bilateral ureteral obstruction was settled, although there was no clear indication of surgeon negligence since the obstruction was thought to be scarring from pre-existing endometriosis.

Risk factors for ureteric injury in all reviewed cases included: obesity (7 cases), previous laparotomy (7 cases), presence of adhesions (6 cases), presence of a large mass/uterus (5 cases) and significant intraoperative bleeding requiring suturing (3 cases).

Since all these cases were associated with potential or actual litigation, we describe below the areas of the records reviewed by the experts and considered by the Courts to determine the standard of care and causation aspect associated with these injuries.

## Pre-operative care

### Indications for surgery and alternative therapies:

Although the indications for surgery and alternative therapies are important determinants of the standard of care in the preoperative care of a patient, neither one was raised or considered important in any of the present cases. Documentation that alternatives to surgery were attempted or discussed were mentioned in only seven of the 20 cases.

### Informed Consent:

The question of informed consent was raised in several cases but negligence for a lack of informed consent was not determined in any of them. In some cases, the patient was not explicitly informed about the risk of ureteric injury. In case No. 12, it was determined that the gynaecologist had not properly obtained informed consent from the patient as evidenced by a lack of a specific discussion regarding injury to the ureter or bowel. However, in this case the Judge concluded that the patient would have proceeded with a hysterectomy even if she knew of the risks of ureteric injury because of the objective test; ‘a reasonable person with the same symptoms would have proceeded with surgery’ in accordance with Canadian law.

A 2015 ruling in the United Kingdom (Montgomery vs Lanarkshire Health Board [2015] UKSC 11), redefined the standard for informed consent and disclosure. The test of materiality defined in the Montgomery ruling was whether “a reasonable person in the patient’s position would be likely to attach significance to the risk, or the doctor is or should reasonably be aware that the particular patient would be likely to attach significance to it”. This means that the doctors must provide information about all material risks; they must disclose any risk to which a reasonable person in the patient’s position would attach significance and enable the patient to use it meaningfully (Chan et al., 2017). The significance of the Montgomery ruling in UK remains to be determined in other countries including Canada.

### Lessons:

Informed consent should be specific and well-documented, and it should include risks, benefits, and alternative therapies. In Canadian courts, negligence for a lack of informed consent has not been a major problem since a reasonable person would consent to the procedure even if all of the potential risks were provided.

## Intra-operative care

### Intraoperative ureter identification and/or urologic consultation:

In one of the 9 laparoscopic hysterectomy cases (case No. 11), one expert opined that the failure to visually identify the ureter or dissect it prior to transecting pedicles, constituted a breach of the standard of care. In a previous publication, we described how we regularly teach learners to identify the ureter at the pelvic brim, during all laparotomic procedures. Invariably, the ureters can be traced from the pelvic brim down to within approximately one centimetre of where it passes under the uterine artery. Further dissection itself may result in inadvertent major complications, such as bleeding from the major vessels or devascularisation of the ureter which may lead to ureteral necrosis, narrowing or obstruction from fibrosis (Vilos et al., 1999).

In most of the laparotomic cases, it was explicitly stated in the operative notes or at the Examination for Discovery (also referred to as Deposition) that the ureters were identified either by visualization, palpation, or identification of ureteral peristaltic activity, either at the beginning or the conclusion of the surgery. In case No. 18, the gynaecologist was found to be negligent for not identifying the ureter (either visually or by palpation) prior to dissecting a retroperitoneal cyst. In contrast, case No. 20 is an example where identification of the ureter was used against the defendant gynaecologist; where the question raised by experts was: If the ureter was isolated at the infundibulopelvic ligament, why was it clipped?

In case No. 17, there were dense bowel adhesions and endometriosis scarring that made it more difficult/impossible to identify the ureters. This case ended with a settlement – likely because of the notion that the gynaecologist should have sought intraoperative urology assistance due to the high risk of injury due to adhesions.

### Lessons:

- In the absence of increased risk factors for ureteric injury during surgery, the expectation is that surgeons rely on their “invariable practice” of identifying ureteral integrity in some fashion (whether by visualization, palpation) at the start and/or at the conclusion of surgery. What constitutes ureteral identification as a routine practice may be different among individual surgeons.
- If there are risk factors that increase the risk of injury (including a retroperitoneal cyst, the ureter running close to a pedicle, an elevated BMI, double ureters, dense pelvic adhesions or excessive bleeding requiring additional suturing), attempts should be made to identify the ureter intraoperatively (ideally through visualization, palpation, or through expert assistance).
- Intraoperative urologic/colleague consultation should be considered if there is concern for ureteral involvement in the surgical field (such as deep infiltrating endometriosis). However, the decision to do so is left up to the clinical judgement of the gynaecologist depending on their level of comfort and experience in performing complex pelvic surgery.

## Steps to prevent and/or identify ureteral injury

### Pre-operative stenting of the ureters:

In complicated cases, stents may be helpful in the identification of the ureters by palpation during laparotomy or vaginal surgery or by visualization if using lighted/ flashing stents during laparoscopy. However, the use of stents may provide a false sense of security and also may decrease the natural mobility of the ureters to fall away from the operative field making them more vulnerable to injury (Vilos et al., 1999).

### Intra-operative Cystoscopy:

Several reports have demonstrated an increased rate of intra-operative bladder and ureteral injury detection from a routine use of intra-operative cystoscopy (Vakili et al., 2005; Jelovsek et al., 2007; Gustilo-Ashby et al., 2006). However, others have reported no increase in intra-operative injury detection and no decrease in post-operative injury detection (Sandberg et al., 2012; Teeluckdharry et al., 2015).

In a 2018 systematic review of urinary tract injury in gynaecological laparoscopy for benign indications, Wong et al found that although the use of routine cystoscopy increased the rates of intra-operative detection of ureteral injury from 38% to 53% and of bladder injury from 84% to 94%, neither was a statistically significant improvement (Wong et al., 2018). The authors stated: In concurrence with data from Visco et al. (2001) noting that routine cystoscopy becomes cost-effective only over a ureteral injury incidence of 2% in laparoscopic hysterectomy, our findings do not appear to support routine cystoscopy from a cost-effectiveness viewpoint. However, as pointed out by Gilmour et al.(2005), the Visco et al. (2001) analysis had excluded the associated costs of lower urinary tract injury as a leading cause of litigation for gynaecological surgeons (Gilmour & Baskett, 2005).

Therefore, at present in Canada, most gynaecologists do not perform cystoscopy during pelvic surgery even if the surgery was difficult and the risk of bladder or ureteric injury was increased. The reason for this is that acquiring cystoscopy skills is not part of the Obstetrics and Gynecology residency curriculum for Royal College of Physicians and Surgeons of Canada (RCPSC) certification and most residents graduate from their obstetrics and gynaecology training without learning to perform a cystoscopy and do not feel comfortable using this basic and fundamental diagnostic instrument; consequently, its use is not the standard of care expected of an average Canadian gynaecologist. In addition, the Society of Obstetricians and Gynecologists of Canada (SOGC) has no clinical practice guidelines as it relates to the indications and application of cystoscopy.

Although routine cystoscopy requires some additional training and expertise and represents an additional surgical procedure with its own inherent risks and complications, it may be time to revisit this important patient safety and contemporary competency curriculum issue, as more of our residents are trained to perform endoscopic procedures once in clinical practice. The American Association of Gynecologic Laparoscopists practice guidelines recommend that minimally invasive gynaecologic surgeons consider the routine use of an immediate post-operative cystoscopy for laparoscopic hysterectomies (AAGL, 2012).

### Lessons:

Pre-operative stenting of the ureters may be helpful in the identification of the ureters in complicated cases. Intra-operative cystoscopy by trained/certified surgeons should be considered in suspected cases of urinary tract injury during gynaecological surgery.

## Post-operative care

### Post-operative period vigilance:

As in this review, most cases of ureteric injury are identified post- operatively (Hove et al., 2010). All 20 incidents were identified post-operatively (with one case of bilateral ureteric injury where one of the injuries was identified intra-operatively). Therefore, surgeons must maintain vigilance in the post- operative period for early signs and symptoms of ureteric injury. Patients from cases No. 4, 7, 8, 9 and 15 were diagnosed with a ureteric injury within 72 hours of having their operation. Of these, only case No. 15 ended with an unfavourable litigation outcome for the surgeon.

Signs and symptoms of ureteric injury included lower back pain, abdominal pain, flank pain, nausea (with or without vomiting), a low-grade fever, ileus, a low urine output, the smell of urine in postoperative drain, and vaginal discharge of serosanguinous fluid. In these cases, the surgeons promptly ordered imaging that confirmed a ureteric injury and consulted a urologist immediately. As the liberal utilization of ultrasound has become an invaluable tool to evaluate renal and/or pelvic pathology, ultrasonography is also invaluable in the immediate postoperative period to evaluate potential urinary tract injury and it should be performed quite liberally.

## Presentation of ureteral injury

### Immediate Signs & Symptoms:

Recognizing ureteral injury in the immediate post-operative period is often challenging, as symptoms may be non-specific and attributed to the normal recovery process. In general, following uncomplicated surgery, patients should typically be showing a rapid improvement in pain symptoms rather than prolonged pain, which could be a sign of visceral organ injury. Imaging with contrast should be done to determine whether there is spillage of bowel contents or urine into the peritoneal cavity or into the vagina.

### Elevated serum creatinine:

We have previously reported that elevated serum creatinine and blood urea nitrogen (BUN) mimicking acute renal failure after laparoscopic surgery are indicative of urinary tract injury (bladder or ureter) and extravasation of urine (Vilos et al., 2001). In the present series, free urine in the abdomen and pelvis was noted/suspected in 4 cases (Case No. 9, 10, 11, 18); however, only in 3 cases (Case No. 7, 10, 11), the creatinine and BUN were elevated indicating that this finding is not always pathognomonic of ureteral injury, though it should raise suspicion of free urine in the peritoneal cavity.

In 14 cases (70%), ureteric injury was identified after the patient was discharged home. Signs at presentation included abdominal pain, leaking fluid from the vagina, flank pain, fever, peritoneal signs, groin pain and abdominal distension. Most patients presented within the first two weeks of being discharged home, however one patient (case No. 15) presented on POD #38.

### Lessons:

Post-operative care is an important determining factor for averting litigation and negligence as most ureteric injuries are identified in the post-operative phase and after discharge. Although the incidence of inadvertent ureteric injuries is rare, these cases highlight the importance of being vigilant and mindful when considering differential diagnosis, when post-operative symptoms occur.

## Uretal injury may be unavoidable and does not always indicate negligence

Ureteric injury can lead to serious medical consequences with complicated clinical outcomes as described in our review; however, most cases (11/20, 55%) had a favourable legal outcome for the defendant physician. In case No. 4, the judge, guided by expert opinion and evidence-based literature, concluded that there is a less than 1% inherent risk of ureteric injury in gynaecological surgery and that in itself, does not imply negligence – provided there is proper pre-operative planning, safe intra-operative conduct, and vigilant postoperative care.

Ureteral injuries occur across a range of pathological conditions, operators and operative techniques which suggests that there may be a critical incidence of ureteral injury below which gynaecological surgery has not been able to fall (in the range of 0.02% to 0.4%). This may be because, due to biological variability, the exact position of the ureter is not constant. This may be especially true in cases of pelvic pathology such as tumours (fibroids), deep infiltrating endometriosis, adhesions, etc.

The biological variability was demonstrated in a study on 52 unanaesthetised women who had a CT to determine the location of their ureters. The findings indicated that the average distance from ureter to the cervical margin was 2.3 ± 0.8 cm (range, 0.1- 5.3 cm). There was no relationship with age, but there was a linear relationship between this distance and body mass index (R2 = 0.075; P = .049); thus, the ureter was slightly more proximal to the cervical margin in heavier women. The authors concluded: In women with apparently normal pelvic anatomy, the average distance between the ureter and cervix is >2 cm. The finding that this distance is <0.5 cm in 12% of the women studied may explain the relatively common occurrence of ureteral injury during hysterectomy. The relationship between body mass index and location is clinically insignificant (Hurd et al, 2001). Therefore, it is well recognized that ureteral damage can occur even when the requisite care and standard precautions and steps are taken by the surgeon to avoid injury to the ureter (Hurd et al, 2001; Dwyer, 2010).

## Future directions

### Use of different energy sources during all gynaecological procedures:

In the 3 cases of ureteral injury associated with a linear stapling device, experts opined that there was no breach of the standard of care while in 4 of 6 cases where bipolar electrosurgery was used, experts opined that the standard of care was breached, likely by inappropriate application of bipolar electrosurgery. This observation raises the question of inadequate training and/or a misunderstanding of the use and safety of electrosurgery in general gynaecological surgery and during endoscopic procedures specifically. It has been well documented that inadequate training and a lack of knowledge of electrosurgery and other newer energy sources is lacking and quite prevalent among learners and practicing surgeons in all disciplines and specialties (Ha et al, 2018).

Since the use of different energy sources is now well incorporated into most gynaecological procedures (vaginal, open, endoscopic robotic), it is imperative that teaching curricula and clinical practice guidelines be developed on the safe application of electrosurgery and other energy sources during surgery and that privileges be accorded after documented competence is evidenced; especially when new emerging technologies are introduced.

### Lesson:

There is a need for formal training programmes in surgical energy and emerging vessel sealing technologies for operators and theatre staff alike, to provide safe surgery.

### Establish Clinical Practice Guidelines:

We recommend that corresponding societies, such as the Society of Obstetricians and Gynecologists of Canada, the American College of Obstetricians and Gynecologists, and others, develop clinical practice guidelines especially on intra-operative identification and/or dissection of the ureter and the practice of routine intra-operative cystoscopy. The availability of practice guidelines is particularly important in all litigated cases, as it is the opinions provided by expert medical consultants, obtained by both litigant and defendant lawyers which the courts heavily depend upon to arrive at a judgement. Since these opinions are obviously opposed to each other, the standard of care may be established only by a clinical practice guideline reflecting best practices based on the contemporary, available published evidence at the time of the event.

## Limitations of our view

This review was unable to evaluate important surgeon characteristics such as training, experience, knowledge of technology used, surgical volume, and location of practice (teaching versus non- teaching centre), all variables that may or may not significantly affect the incidence of injury and patient safety. Although the standard of care and the causation aspect of ureteral injuries may be universal, the findings from this study may not be applicable to all countries and jurisdictions.

## Conclusion

Pre-operative care including sound surgical indications, the provision of alternative therapies and informed consent are important. Informed consent should be specific and well documented, with regard to risks, benefits, and alternatives to surgery.

The conduct of surgery was an issue in several cases; as the failure to identify the ureter was particularly important in all laparotomic cases; although in 3 cases, in which it was stated that the ureters were identified, but were injured, resulted in an unfavourable legal outcome for the surgeon.

Intra-operative urologic/advanced gynaecological consultation and ureteral identification should be considered if there is concern for ureteral involvement in the surgical field. Failure to request surgical help, especially when intra-operative difficulty is encountered, poses a high risk for constituting negligence.

In the immediate post-operative period, presenting signs and symptoms including lower back pain, abdominal pain, flank pain, nausea with or without vomiting, bladder dysfunction, a low-grade fever and elevated creatinine should be investigated.

The majority of ureteral injuries were diagnosed after discharge. Post-operative care is one of the most important determining factors for negligence and litigation, as surgeons are expected to consider ureteric injury in their differential diagnosis for post-operative symptoms that are out of keeping with the expected recovery course.

Delayed diagnosis resulted in more severe clinical sequalae and unfavourable legal outcomes for the surgeon. Surgeons should maintain a high degree of vigilance for ureteric injury in these patients - especially if patients re-present to hospital after initial discharge. Early imaging to rule out a visceral organ injury is recommended.

Ureteric injury, in itself, does not constitute negligence, if it is demonstrated that the surgeon provided reasonable care, which would be expected of him/her during the pre-operative, intra-operative and post-operative phase.

We suggest that teaching curricula be developed on the safe use of all energy sources and vessel sealing technologies during open, vaginal and endoscopic surgery; it is clearly important to develop local and national guidelines and establish standards of care to prevent, identify and manage ureteral injury for all gynaecological surgery to raise awareness, avoid further inadvertent injuries and avoid unnecessary medico-legal exposure.

Finally, one must be mindful that patients often litigate not because an incident occurred, but because of a perceived or real lack of empathy and miscommunication between the team of health care providers and the patient and family.
